# Phylogenomic Analyses Indicate that Early Fungi Evolved Digesting Cell Walls of Algal Ancestors of Land Plants

**DOI:** 10.1093/gbe/evv090

**Published:** 2015-05-13

**Authors:** Ying Chang, Sishuo Wang, Satoshi Sekimoto, Andrea L. Aerts, Cindy Choi, Alicia Clum, Kurt M. LaButti, Erika A. Lindquist, Chew Yee Ngan, Robin A. Ohm, Asaf A. Salamov, Igor V. Grigoriev, Joseph W. Spatafora, Mary L. Berbee

**Affiliations:** ^1^Department of Botany, University of British Columbia, Vancouver, British Columbia; ^2^NITE Biological Resource Center (NBRC), National Institute of Technology and Evaluation, Chiba, Japan; ^3^DOE Joint Genome Institute, Walnut Creek, California; ^4^Department of Botany and Plant Pathology, Oregon State University

**Keywords:** carbohydrate active enzymes, evolution, fungal phylogeny, geological time, Gonapodya, pectinases, streptophytes

## Abstract

As decomposers, fungi are key players in recycling plant material in global carbon cycles. We hypothesized that genomes of early diverging fungi may have inherited pectinases from an ancestral species that had been able to extract nutrients from pectin-containing land plants and their algal allies (Streptophytes). We aimed to infer, based on pectinase gene expansions and on the organismal phylogeny, the geological timing of the plant–fungus association. We analyzed 40 fungal genomes, three of which, including *Gonapodya prolifera*, were sequenced for this study. In the organismal phylogeny from 136 housekeeping loci, *Rozella* diverged first from all other fungi. *Gonapodya prolifera* was included among the flagellated, predominantly aquatic fungal species in Chytridiomycota. Sister to Chytridiomycota were the predominantly terrestrial fungi including zygomycota I and zygomycota II, along with the ascomycetes and basidiomycetes that comprise Dikarya. The *Gonapodya* genome has 27 genes representing five of the seven classes of pectin-specific enzymes known from fungi. Most of these share a common ancestry with pectinases from Dikarya. Indicating functional and sequence similarity, *Gonapodya*, like many Dikarya, can use pectin as a carbon source for growth in pure culture. Shared pectinases of Dikarya and *Gonapodya* provide evidence that even ancient aquatic fungi had adapted to extract nutrients from the plants in the green lineage. This implies that 750 million years, the estimated maximum age of origin of the pectin-containing streptophytes represents a maximum age for the divergence of Chytridiomycota from the lineage including Dikarya.

## Introduction

Close associations with plants or plant products characterize the majority of modern fungi. As decomposers of plant materials, fungi in Dikarya (Ascomycota and Basidiomycota) are key players (Floudas et al. 2012). In contrast to the Dikarya, the lineages that diverged early in fungal evolution, such as the zoosporic chytrids and some of the zygomycetes, use diverse sources of nutrition (Stajich et al. 2009). Some are associated with plants, but many are associated with animals or animal products. The diversity of nutritional modes among early lineages raises the questions of what the ancestral food of the fungi might have been, and when the close and ecologically vital association of fungi with plants may have originated (James et al. 2006; Sprockett et al. 2011).

We approached questions about the ancestral nutrition of fungi through analysis of the genes for fungal digestive enzymes that break down plant material. Traditionally, studies on fungal degradation of plant materials have been focused on the breakdown of cellulose or lignin. Cellulose, however, is too widely distributed across organisms including green, red, and brown algae (Popper et al. 2011) for cellulases to serve as good markers for an association with the land plant lineage. As the earliest land plants lacked lignin (Popper et al. 2011), lignin degrading enzymes may not have been part of ancestral fungal toolkit for breaking down plant material (Floudas et al. 2012). Pectins, on the other hand, are polysaccharides found only in cell walls of the Streptophytes, the land plants, and their closest relatives, the streptophyte algae (Sørensen et al. 2011; Wickett et al. 2014). More specifically, pectin-specific polysaccharides and the genes for their synthesis have only been identified in the land plants and in the “advanced” streptophyte algae but not in the early-diverging streptophyte algal species (Sørensen et al. 2011; Mikkelsen et al. 2014). Therefore, enzymes found in fungi that degrade multiple pectic molecules are potentially good indicators of the association between fungi and the land-plant lineage.

In contrast to cellulose and lignin, pectins are water soluble and form a jelly-like matrix in the primary cell walls of plants. Pectins are complex polymers, often with poly- or oligosaccharide (e.g., arabinogalactan, b-1,4-galactan) side chains decorating backbones of homogalacturonan, xylogalacturonan, rhamnogalacturonan I, and rhamnogalacturonan II. Complete degradation of the pectin complex requires multiple enzymes from different families of glycoside hydrolases (GHs), polysaccharide lyases (PL), and carbohydrate esterases (CE) (van den Brink and de Vries 2011; Benoit et al. 2012). Among the pectinases, at least nine families are pectin specific, including three GH families (GH28, GH53, and GH93), four polysaccharides lyase families (PL1, PL3, PL4, and PL11), and two carbohydrate esterase families (CE8 and CE13). Enzymes from GH28 play an important role in the degradation of pectin backbones by fungi (van den Brink and de Vries 2011). The other major nonpectin-specific families, which may also breakdown other molecules, include GH2, GH35, GH43, GH51, GH54, GH78, GH88, GH105, PL9, CE12, and CE1. In this study, we first surveyed the distribution of families of pectinase genes across fungi and outgroups. We then reconstructed pectinase phylogenies for a detailed picture of enzyme diversification during the evolution of major fungal lineages.

Our goal was to reconcile periods of expansion of pectinase genes with the fungal organismal phylogeny to identify times when ancestral fungi were adapting to using plants for nutrients. If early fungi were associated with land plants and the pectin-containing charophytes, the ancestral fungal species would, we predict, have possessed at least some pectinases. If the early fungi fed on animal or animal-like material and became associated with plants only later, the pectinases may not have diversified until more recent cladogenesis and the rise of the Dikarya.

To infer patterns of pectinase evolution among fungi, we needed a robust fungal phylogeny. Despite recent efforts at resolution (James et al. 2006; Liu et al. 2009; Ebersberger et al. 2012), the branching order for the earliest splits among fungi remains controversial. Fortunately, in recent years, a large number of fungal genomes have been sequenced and annotated, leading to phylogenies with increasingly rich sampling of loci and taxa (Ma et al. 2009; James et al. 2013; Suga et al. 2013; Tisserant et al. 2013). In this study, in collaboration with Joint Genome Institute, we initiated whole-genome sequencing of *Gonapodya prolifera, Coemansia reversa,* and *Conidiobolus coronatus.* With an emphasis on the taxa representing early splits in fungi, we sampled whole-genome data from 27 fungi, as well as 13 species from other eukaryotic groups. We applied multiple analytical methods to individual and concatenated genes to explore factors contributing to phylogenetic conflicts and to phylogenetic resolution.

With the improved genomic and taxonomic sampling of fungi and their relatives, and with the study of fungal pectinases, we aim in this study 1) to improve our understanding of higher level fungal phylogeny, 2) to infer the origin and diversification pattern of pectinases among fungi, and 3) to infer, based on patterns of fungal pectinase gene expansion, the phylogenetic and geological age of plant/fungus association.

## Materials and Methods

We sampled a total of 40 species in this study, including 27 fungi and 13 outgroup species. As detailed in supplementary materials and methods, Supplementary Material online, we generated new whole-genome data from *C. reversa* (NRRL 1564)*, Con. coronatus* (NRRL 28638), and *G. prolifera* (JEL 478) to add to data from previously sequenced genomes (supplementary table S1, Supplementary Material online). Assembly statistics are summarized in supplementary table S2, Supplementary Material online.

### Orthology Assignment and Dataset Generation

Orthologous clusters were identified across the 40 different genomes using the pipeline Hal (Robbertse et al. 2011). Briefly, orthologous clusters were identified across a range of inflation parameters (1.2, 3.0, 5.0) using the Markov Cluster Algorithm. These clusters were then filtered using custom Perl scripts to retain only single-copy clusters, that is, one protein per genome, with a minimum representation of 50% of the genomes and to remove any redundant clusters. Unique single copy clusters were further filtered to retain only those where reciprocal best BLAST hits were to and from proteins within a respective cluster.

In this, as in subsequent analyses of pectinase families, we aligned the amino acid sequences using MAFFT L-INS-i (Katoh et al. 2002; Katoh and Standley 2013). Poorly aligned regions of each gene were masked using Alicut v2.0 (Kück 2009) after detection with Aliscore v2.3 (window size = six) (Misof and Misof 2009; Kück et al. 2010; Meusemann et al. 2010). The concatenated matrix “All-w6,” produced by FASconCAT v1.0 (Kück and Meusemann 2010), included 40 taxa and 28,807 amino acid sites from 136 genes. To evaluate the phylogeny in the absence of remotely related outgroups, we deleted all the nonopisthokont species to create the “Opisthokonts matrix.” To evaluate the effect of missing data, we optimized data density with MARE v.0.1.2 (Meyer and Misof 2010), using the All-w6 matrix as input and creating the new alignment “MARE matrix” as the output. For details about matrices used to test the effects of removing outgroups and altering data density, see supplementary materials and Methods, Supplementary Material online.

### Inference of Species Phylogeny

We inferred a maximum likelihood (ML) tree from the concatenated data in the All-w6 matrix and used this as the species tree in subsequent dating and reconciliation analyses. For all analyses of single genes, concatenated genes, and enzyme gene families, we used ProtTest v.3.2 (Darriba et al. 2011) to find the best amino acid substitution models, followed by ML analysis with RAxML ver. 8.0.0 (Stamatakis 2006; Stamatakis 2014) and Bayesian analysis with MrBayes 3.2 (Ronquist et al. 2012). For the species phylogeny with the All-w6 matrix, we used ten independent ML searches with the LG + GAMMA model and 500 “thorough” bootstrap pseudo-replicates with RAxML. We repeated Bayesian analysis of the All-w6 matrix three times, each time with two independent four-chain runs, sampling over 3,000,000 generations.

We tested for rapid radiation during early fungal evolution using the Bayesian polytomy proposal approach suggested by Lewis et al. (2005) and implemented in P4 (Foster 2004). We used the “recodeDayhoff” option to speed analysis by collapsing amino acids into six groups, reducing the size of the All-w6 alignment. We used three settings for each dataset: no polytomy considered or polytomies allowed with a prior probability of 1.0 and a log C constant set either to 1 or 2*.* We used four individual one-chain Markov chain Monte Carlo runs for each setting, instead of a single four-chain Markov chain Monte Carlo run, to reduce the computation time while still allowing us to detect analyses that were locally trapped in suboptimal tree space.

### Survey of Conflicting Signals among Individual Genes

We anticipated that some genes would provide conflicting phylogenetic signals due to horizontal gene transfer, incomplete lineage sorting, or other factors in their evolutionary history. To evaluate the potential conflicts, we applied Four-cluster Likelihood Mapping analysis (FcLM) (Strimmer and von Haeseler 1997) and the Approximately Unbiased test (AU test) (Shimodaira 2002) to nodes of special interest involving the placement of *Rozella,* Blastocladiomycota, zygomycota II, and *Rhizophagus* (supplementary table S3, Supplementary Material online). All the FcLM analysis and AU tests were based on the individual gene alignments with only opisthokont taxa included. To further explore the relative branch support from the data and identify problematic nodes in the phylogeny, we applied random addition concatenation analysis using RADICAL (Narechania et al. 2012). If support for a node was broadly distributed across genes, then the same node should appear in analyses of random samplings of small subsets of genes. Conversely, if genes on average provided weak support for a node, large subsets of genes would be needed for consistent recovery of the node. In RADICAL analysis, the fixation point for a node is the smallest number of genes needed to consistently recover the node in all the best trees. Further details about tests for conflicting signal are given in supplementary materials and methods, Supplementary Material online.

### Enzyme Data Retrieval and Analysis

We surveyed a total of 20 enzyme families that are involved in pectin degradation. For each family, we used known enzyme sequences from plants, Chromalveolata, fungi, and bacteria as query sequences in BLAST searches against the whole genomes of the 40 taxa of interest, using an expectation value of *e*^−^^5^. We chose multiple bacterial sequences within each pectinase gene family to represent the diversity of gene sequences, based on the information at the Carbohydrate Active Enzyme site (www.cazy.org, last accessed November 2014) and the Braunschweig Enzyme Database (www.brenda-enzymes.org, last accessed November 2014). The sequences from the BLASTp results were searched against the Conserved Domain Database (Marchler-Bauer et al. 2011), and any sequences without the predicted functional domains were removed from the analysis. After alignment of gene family sequences, we performed ML searches and 100 ML bootstrap pseudoreplicates and Bayesian analysis with over 10,000,000 generations and with final effective sample sizes over 1,000.

We applied three alternative approaches to reconcile the gene phylogenies with the species tree. Two approaches to reconciliation of pectinase phylogenies with the phylogenomic species tree involved parsimony-based reconciliations with Notung 2.8 (Durand et al. 2006; Vernot et al. 2008; Stolzer et al. 2012). To accommodate uncertainty in the gene phylogenies, we allowed the branches with <70% ML support in the ML to be rearranged to their most parsimonious position (Notung-rearranged analysis). Second, to retain more of the weak phylogenetic signal from gene trees, for each enzyme family, we averaged estimated gene duplications and copy numbers across the 100 ML bootstrap replicates (Notung-bootstrap analysis). In both analyses, the cost of duplication and loss was set as one. The third approach to reconciliation used the model-based program JPrIME-DLRS (Sjöstrand et al. 2012; Mahmudi et al. 2013) to reconstruct the gene genealogies of each enzyme family while simultaneously mapping the duplication/loss events onto the species tree in a Bayesian framework (see supplementary materials and methods, Supplementary Material online).

### Using the Fungus–Plant Association in Dating Fungal Evolution

The origin of the stem lineage of the pectin-containing Streptophytes is probably more recent than 750 Ma (Douzery et al. 2004; Zimmer et al. 2007; Parfrey et al. 2011). Constraining the split between Chytridiomycota and its sister clade to be 750 Ma, the approximate maximum age of plants with pectin in walls, we estimated the geological ages of fungi in our dataset using BEAST 2.1.3 (Bouckaert et al. 2014) and an uncorrelated log normal molecular clock. We constrained the fungal clades from the ML species tree to be monophyletic. After 7 months, the BEAST analysis completed 126,111,600 generations and Tracer v.1.6 (Rambaut et al. 2007) estimated that sample sizes for all parameters were over 200. We discarded the first 25% of the sampled trees and TreeAnnotator calculated a consensus tree from the remainder.

For an alternative dating method, we applied the penalized likelihood algorithm and Truncated-Newton method implemented in r8s Vers. 1.8 software (Sanderson 2003) (available from http://loco.biosci.arizona.edu/r8s/, last accessed August 2014) to the ML tree that was inferred as described previously. We selected a smoothing parameter value of 3.2 to predict rate changes in our dataset by using r8s's cross validation routine. Alternative algorithms and methods gave similar results and all replicates using the same algorithm and method gave identical results.

### Growth Tests of *Aspergillus niger, Allomyces sp.*, and *G. prolifera*

We tested growth of *A. niger* (CCCM 872)*, Allomyces* sp (CCCM 025)*,* and *G. prolifera* (JEL 478) using carbon sources from two pectic sugars, polygalacturonic acid (PGA; Sigma Aldrich, St. Louis, MO, Cat. no. 81325) and rhamnogalacturonan I (RGI; Megazyme, Bray, Ireland, Cat. no. P-RHAM11), which are the major components of pectin backbones. The fungi were initially grown on a low carbon solid medium of ¼-mPmT (0.01 g Tryptone and 0.01 g peptonized milk powder in 100 ml H_2_O). We cut 5.5 mm mycelial plugs and transferred each into a flask with 30 ml of one of four different liquid media, ¼-mPmT, ¼-mPmT + glucose, ¼-mPmT + PGA, and ¼-mPmT + RGI, using three replicates per medium. The flasks with *G. prolifera* and *Allomyces* sp*.* were incubated at room temperature for one month. Those with *A. niger* were incubated for 11 days. We photographed the fungi and measured the diameter of the mycelial ball surrounding the initial inoculum in each flask.

## Results

### Higher-Order Relationships within Fungi

**Fig. 1.—:**
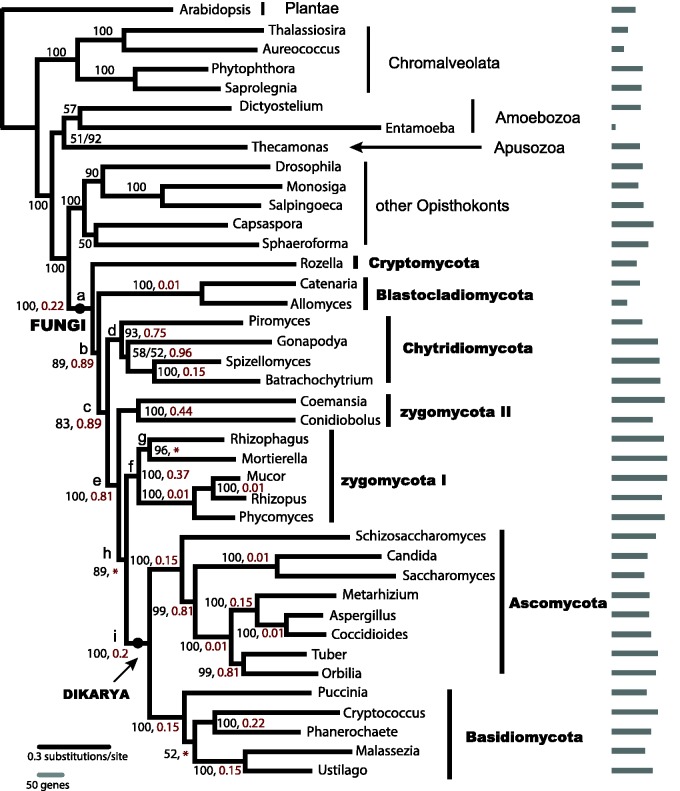
Phylogeny of fungi based on analysis of data from 40 genomes. Numbers in black are the bootstrap values from ML analysis; the posterior probability from Bayesian analysis was one unless specified otherwise by a numeral following a slash. Numbers in red are the fixation points in RADICAL analysis, and smaller numbers indicate better support for the node, whereas red asterisks indicate nodes so poorly supported that they were not fixed. The sidebars show the number of genes present in each species, out of the 136 genes included in the analysis. *Mucor circinelloides* and *Mortierella verticillata* had the largest number, 126 genes each.

The phylogenetic relationships among major fungal groups were largely consistent across different analyses in this study, with moderate to strong support (fig. 1, supplementary table S4, Supplementary Material online). The monophyly of fungi was well supported. *Rozella* represented the earliest split from the remaining fungi, followed by the two Blastocladiomycota taxa, *Allomyces* and *Catenaria.* Chytridiomycota formed a clade sister to predominantly terrestrial fungi consisting of zygomycota I and zygomycota II, Ascomycota, and Basidiomycota. In our dataset, out of all the 136 single-copy orthologs used in the phylogeny reconstruction, only 38 genes were found in *Allomyces,* 60 in *Rozella*,** and 66 in *Catenaria,* whereas the other fungal species averaged 100 genes present (fig. 1). The relationships among these taxa that were missing the most data were also among the least well supported in our analyses.

The two P4 analyses that allowed the proposal of polytomies during tree searches (P4 analysis polytomy 1 and polytomy 2 in supplementary table S4, Supplementary Material online) only weakly supported *Rozella*'s divergence at the first split in fungal evolution. Of the 34 genes that could be used for the FcLM test for the placement of *Rozella*, none showed majority support for any candidate topology (supplementary fig. S1 and table S5, Supplementary Material online). Similarly, in the FcLM tests for the placement of Blastocladiomycota or zygomycota II, only one or two genes showed majority support for a specific topology (supplementary table S5, Supplementary Material online). The results of AU tests were consistent with those of FcLM test (supplementary table S6, Supplementary Material online) in that the majority of genes showed no preference for any one of the candidate topologies.

In the RADICAL analysis, all nodes within fungi were fixed except for nodes h and g, and one node within Basidiomycota, labeled with asterisks in figure 1. Consistent with expectations, the percentage of genes needed for fixation was low for nodes with high support from ML bootstrapping or from Bayesian posterior probabilities. Half of the nodes were fixed with sampling of less than 30% of the total number of genes. On the other hand, the early splits of the ancestors of the Dikarya from a succession of sister taxa were not consistently recovered in the concatenation process until large numbers of genes were sampled (fig. 1, nodes b, c, e, and h, with fixation point higher than 0.8 or not fixed).

Conflicting results obscured the relationship of *Rhizophagus* to *Mortierella* and the rest of the Mucorales. Although *Rhizophagus* and *Mortierella* were resolved as sister groups with 96% bootstrap support from the complete dataset, their shared node did not go to fixation in RADICAL analysis. All of the 107 genes included in the FcLM analysis showed (in 84.4% or more of the sampled quartets) a strong preference for the *Rhizophagus*–*Mortierella* sister relationship. The FcLM results were based on repeated random selection of only four taxa to represent the quartet of lineages; however, the AU tests involved all available taxa. The difference in taxon sampling may underlie the difference in results; in AU tests, only two genes from the Opisthokonts matrix gave significant results and each supported a different topology. Of the two, one strongly supported *Rhizophagus–Mortierella.* This gene encoded a proteasome stabilizer with an Ecm29 domain. Although it accounted for fewer than 1% of sites, support for the *Rhizophagus–Mortierella* clade dropped from 89 to 50% with its removal from the concatenated “Opisthokonts” matrix that excluded outgroup taxa. In contrast, removal of the other gene that passed AU test had a limited impact on the support value (from 89% to 82%).

The “MARE” matrix, constructed to increase data density (supplementary materials and methods, Supplementary Material online) did not include the proteasome stabilizer gene and it showed 100% bootstrap support for an alternative placement of *Mortierella* and *Rhizophagus,* with *Mortierella* sister to Mucorales (*Mucor, Phycomyces,* and *Rhizopus*) rather than *Rhizophagus.* The proteasome stabilizer gene was present in all the species in zygomycota I but none of the sampled Basidiomycota species. Its absence from one major group of terrestrial fungi raises the possibility that the underlying evolutionary processes may have been different in this gene compared with the others. The combination of the unbalanced taxonomic distribution of one, highly influential gene, with the conflicting results from different analyses suggested that the sister relationship between *Rhizophagus–Mortierella* should be treated with caution until further evidence is collected.

### The Evolution of Pectin-Specific Enzymes in Fungi and Other Eukaryotes

**Fig. 2.—:**
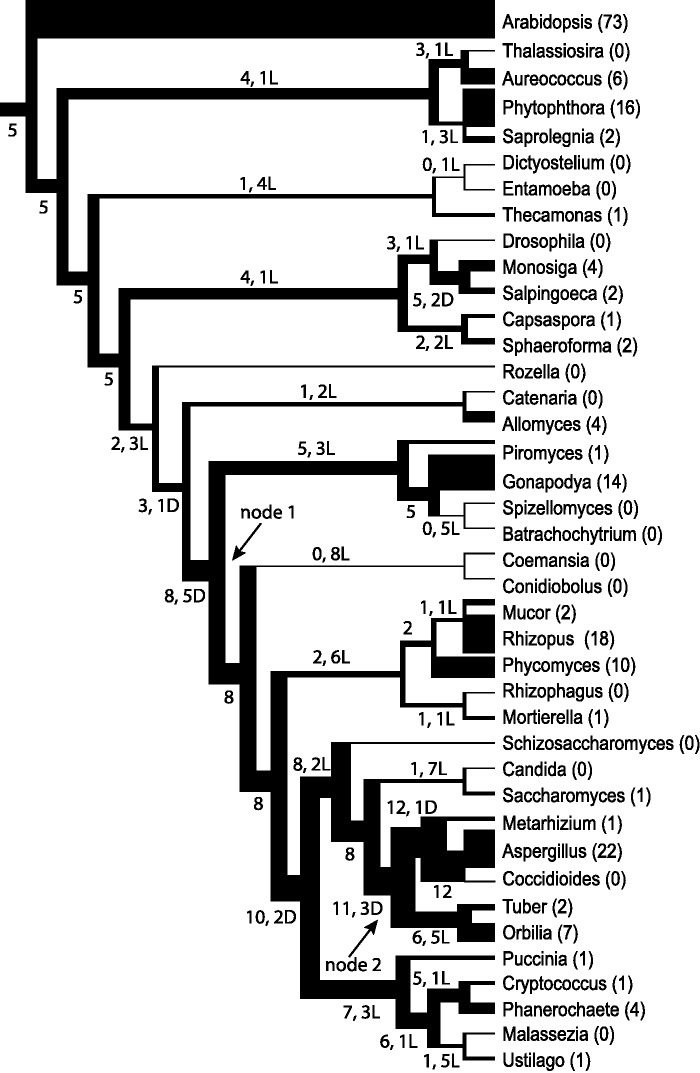
Evolution of GH28 pectinase genes within the phylogeny of fungi, based on Notung-rearranged gene tree/species tree reconciliation. Branch thickness is proportional to pectinase copy number. The numerals above the branches are the copy numbers, followed by the number of duplication (D) or loss events (L) associated with the branch, both estimated by “Notung-rearranged analysis.” Within species, duplication and loss events are not counted. Following the genus name is its total number of GH28 gene copies. Pectinase gene expansions were concentrated on branches leading to node 1, which represents the most recent common ancestor of Chytridiomycota and terrestrial fungi, and to node 2, representing the common ancestor of Pezizomycotina.

**Fig. 3.—:**
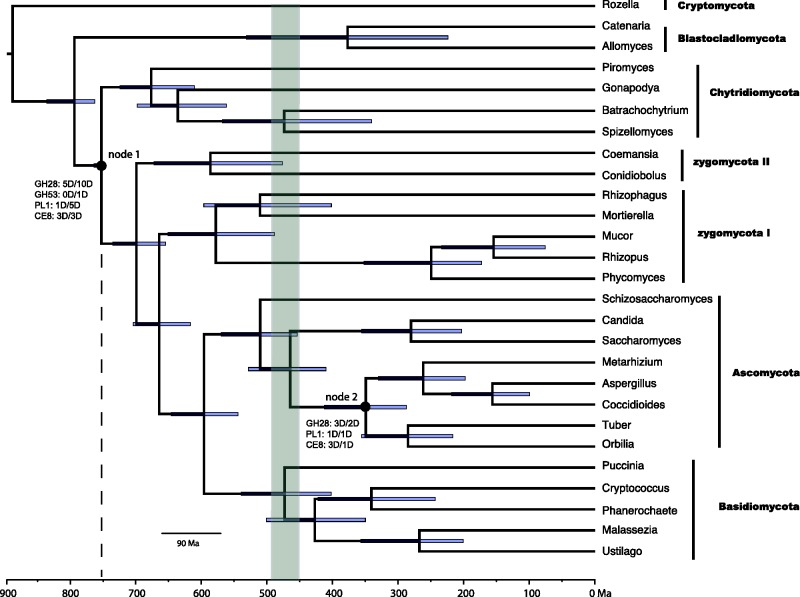
Chronogram showing the effect on fungal ages of assuming that Chytridiomycota diverged from its sister clade only after pectin evolved in plant cell walls, some 750 Ma. Pectinase gene expansions were concentrated on branches leading to node 1, which represents the most recent common ancestor of Chytridiomycota and terrestrial fungi, and to node 2, representing the common ancestor of Pezizomycotina. At these branches, the letters and numbers before a colon designate a pectinase family in which gene duplications mapped to the branch, followed by the number of duplication (D) events estimated from the Notung-rearranged analysis and then from the Notung-bootstrap analysis. The shaded area shows the estimated age of origin of land plants by recent studies (Steemans et al. 2009; Rubinstein et al. 2010).

We inferred that the most recent common ancestor of all fungi possessed one or more copies of pectinases in seven gene families (table 1). Among the fungi, duplication events were concentrated in the common ancestor of Chytridiomycota and the terrestrial fungi (the clade including zygomycota I, zygomycota II, Ascomycota, and Basidiomycota) and in the common ancestor of Pezizomycotina (figs. 2 and 3). Supporting the finding that at least some of its pectinases are functional, *G. prolifera* (Chytridiomycota) grew significantly faster on media supplemented with pectic sugars than on unsupplemented, low carbon media (supplementary table S7, Supplementary Material online). As expected, *A. niger* (Ascomycota) also grew more quickly with pectic sugar supplements.

**Table 1:** Estimated Numbers of Pectin-Specific Enzymes in Ancestral Fungi and Oomycetes

	GH28	GH53*	PL1*	PL3*	PL4*	CE8	CE13
MRCA of all fungi	2/11	3/3	2/9	2/5	4/4	3/7	1
MRCA of all fungi excluding *Rozella*	3/12	3/3	2/9	2/5	4/4	3/7	1
MRCA of Chytridiomycota and *Aspergillus*	8/22	3/4	3/14	2/3	4/4	6/10	1
MRCA of *Phytophthora* and *Saprolegnia*	1/2	1/1	2/4	1/3	1/1	1/1	1

Note.—MRCA, most recent common ancestor. Each cell gives a conservative number estimated by “Notung-rearranged analysis” followed by the more liberal estimate from “Notung-bootstrap analysis.” Asterisks designate enzyme families including one or more bacterial sequences.

The seven different pectin-specific enzymes in the GH28 family (www.cazy.org, last accessed 15 September, 2014) play important roles in breaking the backbones of pectins. In our survey, we found homologs to GH28 enzymes in genomes of 29 eukaryotes including 16 fungi (supplementary table S8, Supplementary Material online; fig. 2). Among the flagellated, predominately nonterrestrial fungi, *Allomyces* had 4 GH28 homologs; *Gonapodya* had 14, and *Piromyces* had 1. The only groups of fungi without homologs were Cryptomycota and zygomycota II. Although the phylogenetic relationships among these GH28 copies were not well resolved, we recovered several fungal-specific clades with strong support (clades A, B, and C in supplementary fig. S2, Supplementary Material online). Each of these fungal-specific clades comprised copies from both the nonterrestrial lineages and the terrestrial lineages. Reconciliation analyses indicated that the earliest fungi had copies of GH28 genes (table 1), which further duplicated during the evolution of the common ancestor of Chytridiomycota and the terrestrial fungi (fig. 2).

The six other families of pectin-specific enzymes found in fungi were also present in other eukaryotes (table 1 and supplementary table S8, Supplementary Material online). The phylogenies for these genes were poorly resolved whether analyzed by ML, Bayesian, or JPrIME-DLRS approaches. Reconciliation methods that produced more highly resolved gene trees resulted in greater numbers of duplications that mapped to deeper branches. Although the two Notung reconciliation analyses showed similar distributions of duplication events, the more highly resolved trees in the Notung-bootstrap analysis predicted higher numbers of duplications along the early branches that predated the split of fungi from other organisms, as well as among ancestral fungi (table 1). JPrIME-DLRS did not report estimates of ancestral copy numbers but it did produce highly resolved Bayesian gene trees, which resulted in still higher numbers of duplication events mapping onto the early branches. When we collapsed nodes with support lower than 0.98 from the JPrIME CE8 family tree and then reconciled the JPrIME tree with the species tree using Notung, the results were similar to estimates from using JPrIME alone, that is, high numbers of duplications mapped to early branches. In comparison, when we collapsed more branches by setting the threshold for retaining a JPrIME branch to 0.999, results converged with the estimation by Notung-rearranged analysis and fewer duplications mapped to early branches. These experiments show that the resolution in the gene tree has a significant effect on the reconciliation and highlight the difficulties involved in reconstructing ancestral copy numbers of rapidly evolving secreted proteins.

Outside of fungi, *Arabidopsis thaliana* (Plantae), with 179 copies of various pectinases, had the greatest number of gene copies. *Phytophthora infestans* ranked second with 84 copies. However, the most recent common ancestor of *Saprolegnia* and *Phytophthora* was reconstructed with only 8 or 13 pectin-specific pectinase genes, and the duplications leading to the high copy numbers were mainly mapped to the terminal branch of *Phytophthora*, instead of the branch leading to the shared ancestor of *Phytophthora* and *Saprolegnia.*

### Age Estimates of Major Nodes in Fungal Evolution

The age estimates from BEAST and r8s were comparable. The origin of fungi was dated as 887 Ma in BEAST and 816 Ma in r8s, which also marks the greatest difference in age estimates between the two programs. The age of the ancestor of terrestrial fungi was 699 Ma in BEAST and 706–718 Ma in r8s analyses (fig. 3). Most of the diversification within terrestrial fungal phyla occurred within the last 500 Ma, based on both analyses.

## Discussion

### Resolving the Early Splits in Fungal Evolution

With new genome sequences for three early diverging fungi, we have contributed to a well-resolved picture of early fungal divergences. For taxa that we have in common, our results are congruent with other studies that have used whole-genome sequences. Early fungal evolution took place among predominantly aquatic, zoosporic lineages, represented by extant Cryptomycota, Chytridiomycota, and Blastocladiomycota (Ebersberger et al. 2012; Torruella et al. 2012; James et al. 2013). Among the terrestrial fungi, the arbuscular mycorrhizal *Rhizophagus*, representing Glomeromycota, is a member of the zygomycota I clade. This is consistent with Tisserant et al.'s (2013) analysis of complete genome sequences, but it contradicts earlier studies based partly or largely on ribosomal gene sequences that showed Glomeromycota to be the sister to Dikarya.

Phylogenomics has proven useful in resolving some controversial phylogenetic problems. However, potential pitfalls remain even with genome-scale data (Fernández et al. 2014). To test for one such pitfall, we looked for conflict among individual genes using the FcLM tests and AU tests. Instead of finding strong conflicting signals, FcLM tests and AU analyses revealed low information content in individual genes. Almost all of the individual genes showed equivocal support for competing topologies at the nodes of interest (supplementary tables S5 and S6, Supplementary Material online). The lack of information content per gene may explain the conflicts among earlier studies based on fewer genes (James et al. 2006; Sekimoto et al. 2011; Ebersberger et al. 2012). It suggests that further resolution of the deeper divergences will come from additional genome sequences of early diverging taxa.

The lack of information content per gene may be due to the nature of these early splits. The earliest splits in fungal evolution happened well over 500 Ma (Taylor and Berbee 2006; Stajich et al. 2009; Parfrey et al. 2011). Any evolutionary signal may have been eroded over time, becoming difficult to recover. Radiation events during early fungal evolution may have been too rapid to allow accumulation of enough substitutions for phylogenetic resolution (Sekimoto et al. 2011; Ebersberger et al. 2012). Results from our P4 Bayesian analysis suggested that a short internode, if not a real polytomy, is present between the first two splits in the fungal tree although not at subsequent nodes. High fixation points or lack of fixation for the earliest splits in RADICAL analysis (nodes b, c, e, and h in fig. 1) also demonstrate the lack of data for resolving these relationships. Similarly, failure to reach fixation in RADICAL analyses reflects difficulty in resolving whether Dikarya is the sister group to zygomycota I, as well as uncertainty about the relationship between *Rhizophagus* and *Mortierella.*

Our experiments with alternative data matrices showed that the branching order of the evolutionarily isolated species of *Rozella, Allomyces,* and *Catenaria* was influenced by gene sequences that were missing from one or more of these taxa. It is unclear whether the loci that are missing from such taxa nevertheless improve overall inferences about their relationships (Lemmon et al. 2009; Wiens and Tiu 2012). Because of the broad taxonomic sampling in this study, relatively few reliably aligned sites were available even for genes that were present, making it difficult to associate substitutions with the short branches of those early-diverging lineages.

### Implications of Pectinase Gene Expansion for Geological Age of Divergence of Fungi

Our analysis showing that the common ancestor of Chytridiomycota and the terrestrial fungi underwent at least nine duplications of pectinase genes points to an ancestral fungal species that was taking advantage of nutrients from pectin-containing streptophyte plants (fig. 3). The only other concentration of pectinase duplications inferred on internal fungal branches consisted of the four or more duplications that mapped to the ancestor of Pezizomycotina (Ascomycota). Supporting their interpretation as clades of orthologs, three fungus-specific clades in the GH28 gene tree containing members of both Chytridiomycota and Dikarya received 89% or better bootstrap support (supplementary fig. S2, Supplementary Material online). Many extant chytrids are associated with streptophyte plants (Sparrow 1960). By showing that the chytrid *G. prolifera* can use the purified pectins rhamnogalacturonan I and polygalacturonic acid as carbon sources (supplementary table S7, Supplementary Material online), we provide evidence of shared pectinase function, beyond homology of the genes and proteins.

Compared with fungi, the fossil record for the plant lineage is rich. By linking fungal evolution to the streptophytes, we provide the first estimate of a maximum age for the fungi. The pectin-containing streptophytes are estimated to be no older than 750 Ma (Douzery et al. 2004; Zimmer et al. 2007; Parfrey et al. 2011) and so the pectin-degrading common ancestor of the Chytridiomycota and Dikarya is probably no older than 750 Ma. Past studies without a constraining maximum age have placed the minimum age origin of fungi and the major terrestrial fungal groups at over a billion years (Heckman et al. 2001; Hedges et al. 2004; Padovan et al. 2005; Parfrey et al. 2011). In addition to being inconsistent with the age of pectin-containing plants, the very old ages for fungi are inconsistent with the notion that diversifications of plants and terrestrial fungi were interdependent on each other (Lücking et al. 2009).

As is clear from the gene trees, proteins homologous to pectinases predate the origin of pectin. Reconstructions suggest at least five copies of the GH28 family were already present in the common ancestor of plants, Chromalveolata, and fungi. The oomycete *Phytophthora* has copies from six pectinase families, and the high copy numbers in each family trace back to recent gene duplications that occurred after it diverged from *Saprolegnia.* The distribution of pectinases across disparate eukaryotes could be the result of horizontal gene transfer from plants or fungi to other organisms, as has been suggested for other genes for secreted enzymes (Richards et al. 2011). It could also reflect phylogenetic error in reconstructing the gene tree (although the methods we used were conservative) or it could reflect diversity of ancestral genes with other specificities that were co-opted for breakdown of pectin.

Pectinases evolve rapidly and have been lost repeatedly from fungi that switched from plant-based nutrition. Yeasts in Dikarya (*Saccharomyces* and *Schizosaccharomyces*) lost all or nearly all of their pectinases as they adopted nutrition from simple sugars (supplementary table S8, Supplementary Material online). Fungi from several lineages lost pectinases when they associated with animals; examples include species of the frog parasitic chytrid *Batrachochytrium*, dandruff causing fungus *Malassezia* and of inhabitants of mucous membranes, *Candida* (supplementary table S8, Supplementary Material online).

Similarly, pectinases appear to have duplicated rapidly in organisms that adopt plant-based nutrition. Our analysis indicates that zygomycete ancestors lost almost all of their pectinase homologs. Although *Rhizophagus irregularis* has no pectin-specific pectinases, the high pectinase copy numbers and low gene diversity of the major pectinase family GH28 in *Phycomyces*, *Rhizopus*,** and *Mucor* represent recent gene duplications, consistent with previous findings (Mertens et al. 2008).

### Microbial Slime and the Origin of Terrestrial Fungi

Early fungi may have moved onto “land” by first living in microbial slime, with mats of streptophyte algae in soil near fresh-water habitats at the edges of rivers or ponds (Gingras et al. 2011; Beraldi-Campesi 2013). Like previous molecular clock dating, our analysis (fig. 3) is consistent with initial Precambrian radiations of terrestrial fungi, more than 100 Ma before the appearance of land plants (Heckman et al. 2001; Parfrey et al. 2011). The earliest lineages of fungi inhabited fresh water, based on the current habitat of *Rozella*, the Chytridiomycota, and the Blastocladiomycota (Sparrow 1960; Karling 1977). Possibly, the ancestors of the first terrestrial fungi followed streptophyte algae from fresh waters onto land, retaining and expanding ancestral enzyme systems to match increasing availability of land plant biomass. Other lineages of eukaryotic multicellular life including Arthropoda and Amoebozoa may have colonized land even before fungi (Schaefer et al. 2010; Fiz-Palacios et al. 2013; Rota-Stabelli et al. 2013; Wheat and Wahlberg 2013), and these inhabitants of soil or other marginal-marine settings (Dunn 2013; Fiz-Palacios et al. 2013) may have nourished the ancestors of the zygomycete lineages, explaining their losses of ancestral pectinases.

Evidence for plant-degrading enzymes in very early fungi supports the idea that fungi lived with the earliest land plants. By solubilizing phosphorus in early terrestrial ecosystems, hyphae of early fungi may have contributed mineral nutrition that supported the first colonization of land by plants (Lenton et al. 2012). Plants in turn contributed to evolution of fungi as well as animals and other kingdoms of terrestrial life (Kenrick et al. 2012). Expansions of multiple pectinase families were associated with the Ascomycota, in the ancestor of Pezizomycotina with an age estimation of 349 Ma, after the Devonian, when land plants diversification promoted the evolution of many other terrestrial lineages.

## Conclusions

Using a robust phylogeny, we show that pectinases, enzymes for degrading plant cell walls, duplicated in an ancestral fungus that probably still lived in freshwater, in association with pectin-containing streptophyte algae. Using the age of streptophytes as a constraint, the age estimate of the common ancestor of terrestrial fungi predates the origin of land plants. Early terrestrial fungi may have evolved in semiaquatic microbial slime, with the ancestors of the zygomycetes tracking arthropods or other animals, while the ancestors of the Dikarya followed plants onto land. Our evidence for an ancient association of fungi with early plants implicates fungi in processes of decomposition and symbiosis in the earliest terrestrial ecosystems.

## Supplementary Material

Supplementary materials and methods, tables S1–S8, and figures S1 and S2 are available at *Genome Biology and Evolution* online (http://www.gbe.oxfordjournals.org/).
